# Tensiomyography-derived contractile parameters in sarcopenic and non-sarcopenic older adults

**DOI:** 10.3389/fragi.2026.1719152

**Published:** 2026-02-04

**Authors:** Katarina Pus, Miloš Kalc, Boštjan Šimunič

**Affiliations:** 1 Institute for Kinesiology Research, Science and Research Centre Koper, Koper, Slovenia; 2 Faculty of Sport, University of Ljubljana, Ljubljana, Slovenia

**Keywords:** muscle function, older adults, sarcopenia, seniors, tensiomyography

## Abstract

**Background:**

Sarcopenia, the progressive decline in skeletal muscle mass and function, is a major public health concern linked to falls, hospitalization and loss of independence among older adults. Initially defined by reduced muscle mass, later also by reduced muscle strength and function, it is now recognized that standard diagnostic tools do not fully capture complexity of sarcopenia. Tensiomyography (TMG) is a non-invasive method that assesses skeletal muscle contractile parameters, which undergo change with aging and sarcopenia. The aims of this exploratory study are to determine whether TMG could be a method for contractile parameters assessment in sarcopenia classification and to evaluate the relationship between TMG-derived parameters and sarcopenia classification tests.

**Methods:**

We included 654 older adults (70.6% women) and included demographics, sarcopenia classification (EWGSOP2, SDOC), muscle strength (handgrip strength, five sit-to-stand), TMG of three leg muscles, muscle mass (bioimpedance), and physical performance (gait speed, timed up-and-go). MANOVA was used to analyze contractile properties and due to low agreement between classifications, we used both classifications. A partial correlation for each sex was conducted to determine the associations between sarcopenia classification tests and TMG-derived parameters of delay time (Td), contraction time (Tc), radial displacement (Dm) and contraction velocity (Vc), controlling for age.

**Results:**

One-way MANOVA confirmed difference between sarcopenic and non-sarcopenic participants according to EWGSOP2 and SDOC classifications in TMG-derived contractile parameters in all three muscles, Td was consistently longer and Dm consistently lower in sarcopenic individuals. Post-hoc univariate tests further demonstrated specific differences due to sarcopenia presence. Age-adjusted partial correlations were weak to moderate, ranging between −0.430 and 0.369.

**Conclusion:**

Sarcopenic individuals exhibited longer Td and Tc, and smaller Dm, though not consistently across all muscles. Td was longer in all three muscles, reflecting electromechnical delays linked to aging. Dm was consistently lower, suggesting increased muscle stiffness. Correlations between TMG parameters and sarcopenia classification tests indicated that shorter Td and Tc, higher Dm, and greater Vc were associated with higher muscle volume, muscle strength and performance. The findings indicate that TMG parameters may be associated with neuromuscular degeneration and sarcopenia, supporting further exploration of muscle- and sex-specific differences.

## Introduction

Sarcopenia, the progressive decline in skeletal muscle mass and function, represents a public health challenge due to its association with an increased risk of falls, frailty, hospitalization and loss of independence among older adults (Cruz-Jentoft et al., 2019). Primarily, sarcopenia was defined as reduced muscle mass, but it has been shown that muscle mass alone does not fully account for the adverse health outcomes such as higher risk of falls and fractures, reduced quality of life and increased risk of all-cause mortality, seen in sarcopenic population (Cruz-Jentoft et al., 2019; Supriya et al., 2021; Ooi and Welch, 2024). Increasing evidence suggests that declines in muscle strength and impairments in muscle’s ability to generate force and sustain physical function play a crucial role in the development of sarcopenia (dos Santos et al., 2017; Beaudart et al., 2025). As a result, the focus of sarcopenia definition has shifted towards broader assessments that include physical performance and strength, recognizing these factors as better predictors of adverse health outcomes than muscle mass alone (Cruz-Jentoft et al., 2019; Beaudart et al., 2025). Despite current definitions including components of muscle strength, muscle mass and physical performance, they rely on arbitrary cut-off points, thereby missing early declines in muscle function. Widely used diagnostic tools, such as hand grip strength test or sit-to-stand test, are indirect and fail to fully capture the nuanced and complex nature of sarcopenia (Supriya et al., 2021). Muscle strength assessments and physical performance tests are influenced by not only quantitative muscle loss but also by neural adaptations, and other factors such as motivation and associated disease, that can make interpretation complex (Clark, 2019; 2023). Notably, muscle strength and physical performance frequently deteriorate prior to declines in muscle mass, suggesting that underlying impairments in muscle contractile properties may preceed muscle mass loss (Tieland et al., 2018). More advanced imaging methods are costly and rarely available outside research settings, which results in a delay in sarcopenia identification. Due to these insights, the revised European Working Group on Sarcopenia in Older People classification (Cruz-Jentoft et al., 2019) has shifted the emphasis from muscle mass to muscle strength, putting muscle strength at the forefront of the classification. This change enhances the need for diagnostic methods that can offer more insight into muscle contractile parameters–not just muscle size–in order to better understand, classify and monitor sarcopenia in clinical practice, as the tools directly assessing these properties remain limited.

Alternative classifications of sarcopenia have been proposed, most recently the Sarcopenia Definitions and Outcomes Consortium (SDOC) (Bhasin et al., 2020) which in contrast to EWGSOP2 defines sarcopenia through a combination of muscle weakness (low grip strength) and low physical performance (low gait speed) without including a measure of muscle mass. These two classifications, EWGSOP2 and SDOC, are based on different conceptual assumptions and have been shown to yield substantially different prevalence estimates on both, global and Slovenian population (Petermann-Rocha et al., 2022; Puš et al., 2025). Evaluating both classifications within the same cohort provides an opportunity to examine how emerging muscle-based assessment methods relate to different operational definitions of sarcopenia and explore whether novel measures of muscle contractile parameters are sensitive to impairments that may be different depending on the classification applied.

Several techniques have been proposed to assess muscle contractile parameters, with electrically evoked mechanical twitches being the most widely used in research settings (Shield and Zhou, 2004). However, this approach presents several limitations. Most notably, it requires specialized dynamometers equipped with force transducers to measure torque at specific joints, making it non-portable and unsuitable for routine clinical application. Additional drawbacks include the time-consuming setup, the requirement for technical expertise, and the restricted applicability to only a few large muscle groups.

Addressing this diagnostic gap requires a sensitive, fast, non-invasive method that directly characterize skeletal muscle contractile parameters. Tensiomyography (TMG) is a mechanomyographic method that allows assessment of muscle mechanical contractile properties directly on the skin above the muscle belly (Valenčič and Knez, 1997; Dahmane et al., 2001; Valenčič, 2001). TMG assesses the radial displacement of the muscle belly due to the thickening of muscle fibers after electrically elicited isometric twitch contraction to extract delay time (Td), contraction time (Tc), half-relaxation time (Tr), peak radial maximal displacement (Dm) and radial contractile velocity (Vc), on several surface muscles. TMG has already been used in older adults population, specifically with master athletes, nursing home residents, patients with knee osteoarthritis and with peripheral artherial disease where it was seen that power athletes and pathological groups showing shortened Tc–which is attributed in power athletes to higher proportion of fast twich fibers due to training regime (Šimunič et al., 2018) and to slow-to-fast shift in muscle fibers due to pathological conditions in chronic hypoxic conditions in leg muscles (McDermott et al., 2020) or regularly found in advanced stage of knee osteoarthritis (Noehren et al., 2018). In contrast, endurance athletes demonstrated prolonged Tc and nursing home residents showing altered Dm patterns indicative of disuse-related changes (Pus et al., 2023). Additionally, it was demonstrated that TMG-derived contractile parameters (Td, Tc and Tr) are strongly correlated (univariately and multivariately) to the VL myosin heavy chain type I (MHC-I) composition (Šimunič et al., 2011) and Dm was found to be negatively correlated with atrophy-related muscle thickness loss after a 35-day bed rest period (Pišot et al., 2008). Moreover, a recent study showed that Dm increased already after a few days of bed rest when ultrasound assessed muscle thickness still remained unchanged (Šimunič et al., 2019) showing great sensitivity of the TMG method to detect early changes in muscle stiffness. However, it remains to be seen if TMG parameters are sensitive to sarcopenia-related contractile parameters changes.

Therefore, the aims of this exploratory study are 1) to determine whether TMG could be a method for contractile parameters assessment relevant to sarcopenia classification and 2) to evaluate the relationship between TMG-derived parameters and sarcopenia classification tests.

## Methods

### Study design

This was a cross-sectional study conducted with older adults across eleven (out of twelve) Slovenian regions between February 2022 and December 2023. Regional health centres were contacted to recruit participants and to provide their facilities to conduct the testing. The study was approved by Slovenian National Medical Ethics Committee (0120-76/2021/6) and was conducted with the principles of the Declaration of Helsinki.

### Eligibility criteria

The inclusion criteria were: a) a minimum age of 60 years and b) signed informed consent.

Exclusion criteria were a) acute illnesses, b) acute fatigue resulting from engagement in strenuous physical activity or prolonged cognitive activity with 24 h prior to testing, c) cancer in terminal stages, d) infections, and e) hospitalisation.

### Procedure

Participants were consecutively recruited between February 2022 and December 2023 across multiple health centres. Each centre had a predefined recruitment period of 1–3 months; in larger centres recruitment was conducted in multiple waves, with new groups of participants enrolled during each period. Participants were invited to join the study via invitation by their chosen general practitioner, and they gave written informed consent at the local health centre. We first collected demographic data (date of birth, body height, body mass), sarcopenia classification measures according to EWGSOP2 – handgrip strength test and five repetition sit-to-stand (5STS), bioimpedance, gait speed and timed up-and-go test (TUG). Physical performance and strength testing was conducted with flexible rest periods with a minimum of 30 s between repetitions and 2 min between tests. Additionally, we collected TMG-derived parameters of three right leg muscles.

### Muscle strength

Two tests are proposed by EWGSOP2 for muscle strength assessment: handgrip strength test and 5STS. Handgrip strength was measured with a hydraulic dynamometer (Jamar, Sammons Preston, USA) in a seated position, dynamometer in a dominant hand, elbow flexed at 90°, and the participants were instructed to squeeze the dynamometer three times with maximal effort. The average of the three attempts was used for the analysis.

The 5STS test was used as a proxy for leg muscle strength. Participants were instructed to stand up from the chair to fully extended position five times as fast as possible with the arms on the chest. Total time was measured with a stopwatch in seconds. For safety reasons, the chair was placed in front of the wall.

### TMG assessment

Contractile properties were assessed with TMG on three muscles on the right leg: postural proximal VL, non-postural biceps femoris (BF) and distal gastrocnemius medialis (GM) due to their involvement in daily movements and known differences in their adaptation to disuse (de Boer et al., 2008). In addition, these are also the most commonly investigated muscles in aging research allowing comparison with previously existing literature. For the assessment of VL, participants were in a supine position with muscles in a relaxed state with knee angle fixed at 30° flexion using a foam pad. For BF and GM, participants were in a prone position, with a 5° knee flexion and neutral ankle position supported with a foam pad. The positioning of the electrodes and a sensor followed previously published protocols (Šimunič et al., 2018) with no prior skin preparation. We included two the most stable parameters, Tc and Dm, in our analysis with the addition of Td and Vc (calculated as Vc = 0.8*Dm/Tc (Langen et al., 2022)).

### Muscle mass or quantity

Muscle mass was estimated with tetrapolar electrical bioimpedance device BIA 101 Anniversary (Akern Srl, Florence, Italy) in a supine position after TMG measures, to ensure adequate lying time (more than 15 min) for reducing stress and minimizing bias due to water distribution. Recorded and used parameters were appendicular skeletal mass and appendicular skeletal mass normalised to squared height.

Where measures of muscle mass were not possible due to mobility issues (*n* = 17), calf circumference was used as a proxy of muscle mass used in sarcopenia classification (cut-off for men was ≤34 cm and for women ≤33 cm).

### Physical performance

Physical performance was assessed with two tests: gait speed over 4 m length and TUG. Two time measuring gates (Beam trainer timing system, Seedgrov d.o.o., Ljubljana, Slovenia) were set over the course of 4 m, participants started 1 m behind the starting gate and were instructed to walk through both gates at the habitual gait speed. The time was measured and the test was repeated twice. Gait speed was calculated for both attempts, and the average was used in the analysis.

TUG test was done for assessment of dynamic balance and functional mobility over the 3 m course. The score consists of the time in seconds needed to stand up from the chair, walk 3 m, pivot around the cone, return and sit down again. We have used standardized chairs of the 45–47 cm height with arm rests. The test was performed twice, and the average time of both attempts was used in the analysis, time was recorded with a stopwatch in seconds.

### Sarcopenia classification

Participants were classified into sarcopenia groups according to EWGSOP2 algorithm (Cruz-Jentoft et al., 2019) and to Sarcopenia Definition and Outcome Consortium (SDOC) algorithm (Bhasin et al., 2020). Distributions of the assessed parameters split by sarcopenia status are provided in the Supplementary Figures S1-S5.

### Statistical analysis

Statistical analysis was conducted in SPSS software (IBM, Chicago, IL, USA), version 29.0.2.0. Multifactor analysis of covariance (MANCOVA) was conducted to determine differences in contractile parameters of BF, GM and VL. The agreement between the two sarcopenia classification algorithms is negative (Fleiss Kappa = −0.825), thus we included analysis using both algorithms. Prior to the main analysis, the assumptions of MANCOVA were tested–normality of each dependent variable was tested with Shapiro-Wilk’s and when violated, appropriate transformation was used. Homogeneity of variance-covariance matrices was examined with Box’s M test and homogeneity of variance was tested with Levene’s test. Multicolinearity was assessed with correlation matrix and acceptable r was below 0.80. The required sample size for MANCOVA was calculated *a priori* using G*Power, based on three dependent variables (Td, Tc and Dm), four groups (non-sarcopenic, presarcopenic, sarcopenic, severely sarcopenic), espected effect size of 0.15. The MANCOVA analyses were conducted separately for each muscle, where EWGSOP2 included four levels (non-sarcopenic, presarcopenic, sarcopenic, severely sarcopenic) and SDOC included two levels (sarcopenic and non-sarcopenic). For each muscle, three TMG parameters (Td, Tc and Dm) were entered as dependent variables in a single multivariate model with age and sex as covariates. Post-hoc tests were conducted for significant MANCOVA results with Bonferroni correction applied to control for multiple comparisons. Lastly, partial correlations were calculated separately for males and females and adjusted for age. The alpha level for all significant decisions was set at p < 0.05 and a partial η^2^ was calculated. Data is presented as means and standard deviations.

## Results

Descriptive statistics of all assessed parameters are presented in Table 1. Our sample consisted of 654 older adults (70.6% female) and the analysis was conducted on the subsample of participants with a complete dataset from which 5.3% (EWGSOP2) and 9.7% (SDOC) were classified as sarcopenic.

**TABLE 1:** Descriptive statistics of assessed parameters.

Parameter (unit)	Pooled data	EWGSOP2		SDOC	
		Non-sarcopenic	Sarcopenic	Non-sarcopenic	Sarcopenic
Age (years)	72.4 ± 8.7 (*n* = 654 (199 M))	70.8 ± 7.3	77.5 ± 10.9	70.0 ± 6.8	81.6 ± 8.0
Body height (cm)	163.6 ± 8.5 (*n* = 638 (196 M))	163.8 ± 8.4	163.4 ± 9.3	164.4 ± 8.2	158.2 ± 8.6
Body mass (kg)	77.0 ± 14.3 (*n* = 638 (196 M))	78.0 ± 14.1	65.4 ± 11.4	77.7 ± 14.2	72.9 ± 14.1
BMI (kg/cm^2^)	28.8 ± 4.78 (*n* = 638 (196 M))	29.0 ± 4.71	24.4 ± 2.92	28.8 ± 4.72	29.10 ± 5.00
ALM/ht^2^	7.88 ± 1.61 (*n* = 623 (192 M))	6.85 ± 0.99	5.72 ± 0.79	6.81 ± 1.01	6.50 ± 1.01
5STS (s)	11.1 ± 4.50 (*n* = 590 (183 M))	10.7 ± 4.08	16.5 ± 7.03	10.4 ± 3.73	17.6 ± 5.64
Hand grip strength (kg)	27.4 ± 11.3 (*n* = 645 (194 M))	29.4 ± 10.5	18.2 ± 7.03	30.1 ± 10.1	15.5 ± 6.69
Timed up-and-go test (s)	8.47 ± 5.91 (*n* = 629 (189 M))	7.17 ± 2.76	10.7 ± 5.02	6.77 ± 1.88	13.2 ± 5.23
Gait speed (m/s)	1.14 ± 0.04 (*n* = 639 (192 M))	1.22 ± 0.33	0.95 ± 0.41	1.27 ± 0.29	0.58 ±. 15

M, males; BMI, body mass index; ALM/ht^2^, appendicular lean mass normalized to squared height; 5STS, five sit-to-stand.

One-way MANCOVA, adjusted for age and sex, confirmed difference between sarcopenic and non-sarcopenic participants according to EWGSOP2 and SDOC classifications in TMG-derived contractile parameters in all three muscles. In VL, using EWGSOP2, we found differences between sarcopenic and non-sarcopenic participants (*F* (9, 1791) = 5.896; *p* < 0.001; Pillai’s Trace = 0.086, partial η^2^ = 0.029) and using SDOC (*F* (3, 589) = 10.257; *p* < 0.001; Pillai’s Trace = 0.05, partial η^2^ = 0.05). In BF, using EWGSOP2, we found differences between sarcopenic and non-sarcopenic participants (*F* (9, 1710) = 4.145; *p* < 0.001; Pillai’s Trace = 0.064, partial η^2^ = 0.021) and using SDOC (*F* (3, 563) = 5.561; *p* < 0.001; Pillai’s Trace = 0.029, partial η^2^ = 0.029). In GM, using EWGSOP2, we found differences between sarcopenic and non-sarcopenic participants (*F* (9, 1746) = 3.527; *p* < 0.001; Pillai’s Trace = 0.054, partial η^2^ = 0.018) and using SDOC (*F* (3, 575) = 7.833; *p* < 0.001; Pillai’s Trace = 0.039, partial η^2^ = 0.039). Post-hoc univariate tests further demonstrated specific differences between sarcopenic and non-sarcopenic in TMG parameters, as shown in Table 2 for EWGSOP and Table 3 for SDOC classifications. Important here is to see, that SDOC classification yielded 25 (83%) more sarcopenic participants than EWGSOP2 classification and that resulted in more differences in TMG-derived parameters between sarcopenic and non-sarcopenic groups.

**TABLE 2:** Multivariate analysis of covariance between different sarcopenia groups, classified by a revised European Working Group on Sarcopenia in Older People classification.

Muscle corresponding TMG-parameter (unit)	Non-sarcopenic	Pre-sarcopenic	Sarcopenic	Severely sarcopenic	F	p-value (η_p_^2^)
*Biceps femoris*	n = 446	n = 99	n = 15	n = 16	​	​
Td/ms	30.8 (4.90)	33.4 (7.44)^a^	31.1 (4.13)	33.9 (6.80)	4.26	0.005 (0.022)
Tc/ms	44.4 (10.6)	45.0 (12.6)	42.3 (11.7)	47.4 (10.8)	0.540	0.655
Dm/mm	7.01 (3.21)	4.81 (2.99)^a^	4.89 (1.92)	5.34 (3.74)	9.66	<0.001 (0.048)
*Gastrocnemius medialis*	n = 450	n = 104	n = 17	n = 17	​	​
Td/ms	23.7 (3.53)	26.0 (6.17)^a^	22.7 (2.51)	26.9 (6.32)^a^	4.38	0.005 (0.022)
Tc/ms	29.9 (7.30)	29.1 (6.90)	29.5 (10.9)	28.5 (5.55)	0.197	0.898
Dm/mm	4.12 (1.87)	2.96 (1.82)^a^	3.28 (1.46)	2.63 (1.50)^a^	6.41	<0.001 (0.032)
*Vastus lateralis*	n = 451	n = 116	n = 17	n = 19	​	​
Td/ms	25.0 (2.58)	27.7 (4.53)^a^	26.4 (2.30)	29.1 (3.40)^a^	9.67	<0.001 (0.046)
Tc/ms	26.0 (4.93)	28.6 (6.95)	27.4 (6.69)	27.3 (4.13)	2.54	0.056
Dm/mm	4.94 (1.85)	3.84 (1.90)^a^	4.02 (1.98)	3.87 (1.58)	8.15	<0.001 (0.039)

^a^ different from non-sarcopenic (p < .001); $ different from non-sarcopenic (p < .01); Td: delay time; Tc: contraction time; Dm: radial displacement amplitude.

**TABLE 3:** Multivariate analysis of covariance between different sarcopenia groups, classified by a sarcopenia definition and outcomes consoritum classification.

Muscle corresponding TMG-parameter (unit)	Non-sarcopenic	Sarcopenic	F	p-value (η_p_^2^)
*Biceps femoris*	n = 512	n = 57	​	​
Td/ms	30.9 (4.96)	34.9 (8.67)	12.9	<0.001 (0.022)
Tc/ms	44.2 (10.8)	46.7 (12.2)	1.21	0.272
Dm/mm	6.70 (3.20)	4.75 (3.38)	5.66	0.018 (0.010)
*Gastrocnemius medialis*	n = 521	n = 60	​	​
Td/ms	23.8 (3.66)	27.2 (6.97)	12.3	<0.001 (0.021)
Tc/ms	29.9 (7.37)	28.2 (6.57)	1.84	0.175
Dm/mm	3.99 (1.88)	2.69 (1.78)	6.66	0.010 (0.011)
*Vastus lateralis*	n = 525	n = 70	​	​
Td/ms	25.2 (2.69)	29.2 (5.60)	23.5	<0.001 (0.038)
Tc/ms	26.3 (5.39)	28.3 (6.00)	0.244	0.621
Dm/mm	4.80 (1.86)	3.66 (1.90)	7.16	0.008 (0.012)

Td, delay time; Tc, contraction time; Dm, radial displacement amplitude.

A partial correlation between TMG parameters and sarcopenia classification tests for males (Table 4) and females (Table 5) was conducted to determine the relationship between sarcopenia classification tests and TMG-derived parameters of Td, Tc, Dm and Vc, controlling for age. Both tables include zero-order and age-adjusted correlations. Correlations were weak to moderate, ranging between −0.430 and 0.369.

**TABLE 4:** Zero order and age-adjusted partial correlations between tensiomyographic parameters and sarcopenia classification tests for males.

TMG-parameter	Correlation coefficient	5STS		TUG		HGS		GS		Muscle quantity	
		Zero order	Age	Zero order	Age	Zero order	Age	Zero order	Age	Zero order	Age
Biceps femoris											
Td	r	0.199	0.128	0.212	0.112	−0.207	−0.105	−0.109	−0.027	−0.153	−0.106
	p	*0*.009*	*0*.*097*	*0*.006*	*0*.*147*	*0*.007*	*0*.*173*	*0*.*156*	*0*.*731*	*0*.042*	*0*.*164*
Tc	r	0.025	−0.012	−0.009	−0.072	−0.137	−0.099	−0.104	−0.071	−0.158	−0.141
	p	*0*.*749*	*0*.*875*	*0*.*907*	*0*.*355*	*0*.*074*	*0*.*202*	*0*.*178*	*0*.*357*	*0*.037*	*0*.*062*
Dm	r	−0.225	−0.178	−0.282	−0.229	0.247	0.188	0.179	0.128	−0.118	−0.151
	p	*0*.003*	*0*.021*	*<0*.001*	*0*.003*	*0*.001*	*0*.014*	*0*.019*	*0*.*098*	*0*.*118*	*0*.046*
Vc	r	−0.177	−0.115	−0.218	−0.135	0.252	0.175	0.200	0.137	−0.031	−0.064
	p	*0*.021*	*0*.*138*	*0*.004*	*0*.*079*	*<0*.001*	*0*.023*	*0*.009*	*0*.*074*	*0*.*685*	*0*.*396*
Gastrocnemius medialis											
Td	r	0.090	−0.019	0.200	0.058	−0.185	−0.040	−0.261	−0.166	−0.123	−0.056
	*p*	*0*.*240*	*0*.*808*	*0*.009*	*0*.*453*	*0*.016*	*0*.*607*	<0.001*	0.031*	*0.103*	*0.458*
Tc	r	−0.152	−0.168	−0.137	−0.168	−0.014	−0.008	0.015	0.023	−0.091	−0.087
	*p*	0.048*	0.029*	*0.074*	0.029*	*0.855*	*0.917*	*0.841*	*0.770*	*0.229*	*0.252*
Dm	r	−0.399	−0.341	−0.430	−0.361	0.214	0.102	0.274	0.197	−0.103	−0.173
	*p*	<0.001*	<0.001*	<0.001*	<0.001*	0.005*	*0.186*	<0.001*	0.010*	*0.172*	0.022*
Vc	r	−0.369	−0.300	−0.410	*−0.323*	0.251	0.130	0.297	0.212	−0.093	−0.170
	*p*	<0.001*	<0.001*	<0.001*	<0.001*	<0.001*	*0.090*	<0.001*	0.006*	*0.222*	0.025*
Vastus lateralis											
Td	r	0.369	0.277	0.354	0.218	−0.334	−0.190	−0.338	−0.235	−0.137	−0.065
	*p*	<0.001*	<0.001*	<0.001*	<0.004*	<0.001*	0.013*	<0.001*	0.002*	*0.070*	*0.391*
Tc	r	0.205	0.124	0.112	−0.025	−0.198	−0.078	−0.193	−0.106	−0.070	−0.020
	*p*	0.007*	*0.106*	*0.144*	*0.749*	0.009*	*0.311*	0.011*	*0.166*	*0.356*	*0.792*
Dm	r	−0.249	−0.208	−0.246	−0.194	0.200	0.138	0.072	0.011	−0.031	−0.062
	*p*	0.001*	0.006*	0.001*	0.011*	0.009*	*0.072*	*0.345*	*0.883*	*0.685*	*0.413*
Vc	r	−0.294	−0.228	−0.255	−0.158	0.220	0.113	0.127	0.038	−0.005	−0.056
	*p*	<0.001*	0.003*	<0.001*	0.039*	0.004*	*0.140*	*0.097*	*0.619*	*0.943*	*0.462*

Td, delay time; Tc, contraction time; Dm, radial displacement amplitude; Vc, radial contractile velocity; 5STS, five sit-to-stand test; HGS, hand grip strength; TUG, timed up-and-go test; GS, gait speed; muscle quantity, appendicular skeletal mass normalized to squared height.

**TABLE 5:** Zero order and age-adjusted partial correlations between tensiomyographic parameters and sarcopenia classification tests for females.

TMG-parameter	Correlation coefficient	5STS		TUG		HGS		GS		Muscle quantity	
		Zero order	Age	Zero order	Age	Zero order	Age	Zero order	Age	Zero order	Age
Biceps femoris											
Td	r	0.167	0.152	0.174	0.160	−0.087	−0.063	−0.237	−0.230	−0.009	0.005
​	p	0.001*	0.003*	<0.001*	0.002*	0.089	0.220	<0.001*	<0.001*	0.858	0.929
Tc	r	−0.005	−0.008	0.013	0.010	0.025	0.031	−0.067	−0.071	−0.205	−0.202
​	p	0.922	0.874	0.796	0.844	0.629	0.545	0.192	0.169	<0.001*	<0.001*
Dm	r	−0.307	−0.244	−0.322	−0.218	0.203	0.106	0.276	0.179	−0.286	−0.340
​	p	<0.001*	<0.001*	<0.001*	<0.001*	<0.001*	0.040*	<0.001*	<0.001*	<0.001*	<0.001*
Vc	r	−0.294	−0.234	−0.304	−0.205	0.183	0.089	0.289	0.201	−0.229	−0.275
​	p	<0.001*	<0.001*	<0.001*	<0.001*	<0.001*	0.083	<0.001*	<0.001*	<0.001*	<0.001*
Gastrocnemius medialis											
Td	r	0.161	0.118	0.196	0.133	−0.186	−0.134	−0.184	−0.126	0.059	0.084
​	p	0.001*	0.020*	<0.001*	0.009*	<0.001*	0.009*	<0.001*	0.013*	0.247	0.099
Tc	r	−0.047	−0.044	0.008	0.021	−0.042	−0.053	−0.045	−0.060	−0.013	−0.021
​	p	0.354	0.385	0.870	0.684	0.413	0.295	0.378	0.241	0.793	0.684
Dm	r	−0.307	−0.244	−0.282	−0.171	0.172	0.071	0.271	0.174	−0.132	−0.171
​	p	<0.001*	<0.001*	<0.001*	<0.001*	<0.001*	0.162	<0.001*	<0.001*	0.010*	<0.001*
Vc	r	−0.307	−0.245	−0.294	−0.189	0.198	0.104	0.308	0.219	−0.145	−0.181
​	p	<0.001*	<0.001*	<0.001*	<0.001*	<0.001*	0.041*	<0.001*	<0.001*	0.004*	<0.001*
Vastus lateralis											
Td	r	0.323	0.234	0.302	0.155	−0.229	−0.108	−0.327	−0.206	−0.070	−0.029
​	p	<0.001*	<0.001*	<0.001*	0.002*	<0.001*	0.033*	<0.001*	<0.001*	0.173	0.571
Tc	r	0.231	0.177	0.210	0.126	−0.182	−0.113	−0.218	−0.145	−0.016	0.004
​	p	<0.001*	<0.001*	<0.001*	0.012*	<0.001*	0.026*	<0.001*	0.004*	0.761	0.935
Dm	r	−0.202	−0.123	−0.318	−0.224	0.169	0.072	0.210	0.103	−0.177	−0.207
​	p	<0.001*	0.015*	<0.001*	<0.001*	<0.001*	0.155	<0.001*	0.042*	<0.001*	<0.001*
Vc	r	−0.256	−0.172	−0.349	−0.241	0.218	0.112	0.256	0.139	−0.175	−0.211
​	p	<0.001*	<0.001*	<0.001*	<0.001*	<0.001*	0.026*	<0.001*	0.006*	<0.001*	<0.001*

Td, delay time; Tc, contraction time; Dm, radial displacement amplitude; Vc, radial contractile velocity; 5STS, five sit-to-stand test; HGS, hand grip strength; TUG, timed up-and-go test; GS, gait speed; muscle quantity, appendicular skeletal mass normalized to squared height.

Among males, the strongest correlation was observed between Dm of GM and TUG (r = −0.430, *p* < 0.001) after being corrected for age (r = −0.361, *p* < 0.001).

Among females, the strongest correlation was observed between Dm of BF and muscle quantity (r = −0.286, *p* < 0.001) after being corrected for age (r = −0.340, *p* < 0.001).

## Discussion

We aimed to explore whether TMG-derived contractile parameters (Td, Tc, Dm and Vc) could be considered as a measure for contractile parameters assessment in sarcopenia classification.

After classifying participants into two groups, sarcopenic and non-sarcopenic, by two proposed classifications, we have found differences between both groups in all TMG-derived parameters; however, not systematically in all three selected muscles. When the difference was significant, sarcopenic group had longer Td and Tc and smaller Dm. Furthermore, we have correlated TMG-derived parameters with all classification tests incorporated in EWGSOP2 and SDOC algorithms and found that Td and Dm are consistently weakly to moderately correlated to sarcopenia classification tests.

### Delay and contraction time

We have confirmed that Td is consistently longer in all three muscles in sarcopenic individuals when compared to non-sarcopenic individuals, regardless of the classification algorithm used. Td measures the electromechanical delay, the time between onset of the electrical stimuli and the mechanical onset of muscle contraction. To date, Td has not been vastly explored among older adults, especially not among the sarcopenic population, but rather in athletes where it was found to be associated with fatigue and training or match recovery (García-García et al., 2019; Fernández-Baeza et al., 2024). Td resembles processes such as the generation of motor nerve action potentials, the transmission of the action potential from nerve to muscle, the propagation of the action potential along the muscle fibers, calcium release and binding and cross-bridges coupling (Miller et al., 2013) which are altered with aging. Consequently, muscle strength and muscle force generation in older adults could be negatively affected by these mechanisms. Literature suggests possible explanations through the instability of the neuromuscular junction (Monti et al., 2021), change of contractile properties and molecular changes in myokine production (Lim and Frontera, 2022). A combination of these alternations could be affecting Td, however more research is warranted.

Results of Tc have been less clear as they did not reveal differences in any of the muscles, when EWGSOP2 classification algorithm was applied. In contrast, when classified with SDOC, which does not include muscle mass measures, longer Tc was shown in VL and as a trend in BF, but not in GM. Prolongation of BF Tc, but not in other muscles, has been previously found also in muscle atrophy after 10 days (Franchi et al., 2022) and 35 days (Šimunič et al., 2019) of bed rest. Tc increases with age, too, in all three muscles in non-athletes and also in master athletes, especially in endurance master athletes after the age of 65 years and this is paralleled to higher proportion of MHC-I, in endurance master athletes (Šimunič et al., 2018). Higher proportion of MHC-I is typically found in aged VL muscle (Tieland et al., 2018), and this is further supported by a decline in the number of type II muscle fibers (Wiedmer et al., 2021), which seems to be more prominent in sarcopenic muscles compared to non-sarcopenic ones (Tanganelli et al., 2021). Another potential explanation for the non-uniform Tc behavior across muscles is that each muscle performs a distinct primary function. Specifically, postural and distal GM could be less affected by sarcopenia than proximal VL whereas BF being least affected as it is non-postural, which is commonly observed also in disuse muscle atrophy (de Boer et al., 2008). In aged population, postural muscles are subject to low-intensity movements, such as standing and balancing and therefore being used in habitual physical activity, while non-postural muscles generate force in faster and stronger propulsive movements.

### Radial muscle displacement

We found that Dm in the sarcopenic group is consistently lower in all three investigated muscles compared to the non-sarcopenic group. A similar trend was confirmed in aged populations in both; a longitudinal (Teraž et al., 2023) and a cross-sectional study (Labata-Lezaun et al., 2023), as well as after unilateral lower-limb amputation (Fujishita et al., 2021). However, the opposite, higher Dm, was found after the bed rest (Pišot et al., 2008; Šimunič et al., 2019). The Dm decrease was speculated to relate to aging changes within the muscle, changes in elasticity of the muscle (Ochala et al., 2007), lower contractile protein density (Prochniewicz et al., 2007) and remodelling of muscle fibers towards less compliant type I. Dm has been shown to relate to muscle stiffness (Macgregor et al., 2018; Šimunič et al., 2019) and based on our findings we can assume that sarcopenic muscle has further increased passive muscle stiffness compared to a non-sarcopenic muscle as it was confirmed for aged population by shear weave elastography (Şendur et al., 2020) as well as in single muscle fibers (Lim and Frontera, 2023), but was not investigated in a sarcopenic cohort. Further, muscle stiffness is related to multiple underlying alternations, such as intracellular changes in myoproteins and/or extracellular matrix (Lee et al., 2020), sarcolemma properties (Garcia-Pelagio and Bloch, 2021) and muscle fat infiltration (Dondero et al., 2024). Impaired stiffness could lead to muscle function impairment, potentially resulting in sarcopenia.

Even though the present study did not look into musculoskeletal imaging, we have found that Dm is sensitive enough to detect differences between sarcopenic and non-sarcopenic groups regardless of the sarcopenia algorithm used.

## A correlation analysis between TMG-related parameters and sarcopenia classification tests

Due to sex differences in several sarcopenia classification parameters, we analyzed the relationship between TMG-derived parameters and sarcopenia classification tests separately for men and women. Moreover, our previous studies showed, that TMG parameters are age-dependent (Šimunič et al., 2018). In the present study, the sarcopenic participants were indeed older than the non-sarcopenic participants (79.9 ± 10.7 years vs. 71.8 ± 3.3 years, respectively); therefore, age-adjusted correlations were computed to allow for better interpretation and discussion of the results.

### Muscle strength

Lower body muscle strength (5STS time) is positively correlated with Td in both sexes (men: VL; women: BF, VL and a trend in GM), negatively with Dm (men: all three muscles, women: BF and GM), and negatively with Vc in both sexes (men and women: all three muscles). Similar correlation between Dm and 5STS was also reported for aged population in rectus femoris and VL muscles by Labata-Lezaun et al. (Labata-Lezaun et al., 2023). Our findings could be interpreted as better lower body muscle strength in those with better neural function, lower muscle stiffness and higher transversal contraction velocity. Interestingly, in the present study, non-invasive TMG-derived VL composition measure (Tc) was also positively correlated with 5STS, in both sexes, suggesting that participants with greater muscle strength may exhibit a higher proportion of type II muscle fibres. This can be supported with the previous findings of Tc parameter being correlated with MHC-I which implied type I muscle fibers (Šimunič et al., 2011). The age-related increase in variability of motor performance appears to involve reduced and more variable synaptic inputs that drive motor neuron activation, fewer and larger motor units, less stable neuromuscular junctions, lower and more variable motor unit action potential discharge rates, and smaller and slower skeletal muscle fibers (Hunter et al., 2016). The aforementioned age-related reorganization of the neuromuscular system can impair the efficiency of neural activation and increase electromechanical delay, which was also observed in our study as an increase in Td. While the correlations between 5STS time and TMG-derived parameters were weak to moderate, we also found several less consistent and weaker correlations between handgrip strength and TMG-derived parameters. Nevertheless, these correlations showed a similar direction, with shorter Td or Tc, higher Dm, and higher Vc observed in participants with higher handgrip strength.

### Muscle quantity

Muscle quantity (defined as appendicular skeletal muscle mass normalized to squared height) is negatively correlated with Tc in the BF of women, with Dm in both sexes (men: VL, women: all three muscles) and also with Vc (men: VL, women: all three muscles). Those with higher muscle quantity have shorter Tc and lower Dm and Vc. This is in agreement with our previous study, where lower muscle quantity was linked to higher Dm also in a longitudinal atrophy study. Specifically, after 35-day bed rest, Dm increased more in individuals with higher muscle atrophy (Pišot et al., 2008). However, lower Dm values were consistently observed across all three muscles in the sarcopenic group, which has lower muscle quantity, compared with the non-sarcopenic group. This pattern appears to be inconsistent with the results of correlation analyses. Taken together, these results suggest the possibility of a non-linear association between Dm and muscle size. This potential inverse u-shaped relationship should be considered in future studies.

### Physical performance

Physical performance assessed by TUG time positively correlated with Td in both sexes (men and women: BF, VL), and negatively with Dm in both sexes (men and women: all three muscles), and Vc in boh sexes (men and women: all three muscles). On the other hand, physical performance assessed by sub maximal self-selected gait speed test negatively correlated with Td in both sexes (men: VL, women: all three muscles), with Tc only in women (VL), positive correlated with Dm in both sexes (men: GM, women: BF, VL) and with Vc in both sexes (men: GM, women: all three muscles). Summarizing both physical performance tests, better performance was found in individuals with shorter Td or Tc, and higher Dm or Vc. Similar results were reported in nursing home residents by Fabiani et al. (Fabiani et al., 2021), who found better TUG performance in those with higher Dm and shorter Tc. Our study added robustness to this correlation with physical performance, as was focused on the general population (aged: 72.45 ± 8.74 years) with a mean TUG time of 8.47 ± 5.91 s, where the study of Fabiani reported data from older (aged: 85.6 ± 7.6 years) less mobile population with a mean TUG time of 20.1 ± 8.1 s. The strength of the correlations between maximal TUG test and TMG-derived parameters were weak to moderate, whereas correlations with submaximal self-selected GS were lower.

This study has certain limitations. While our goal was to recruit a sample representative of the general Slovenian population, there remains a possibility that our participants were skewed toward more active older adults who were more inclined to accept invitations for participation. Nonetheless, we tried to avoid this bias during the initial recruitment phase. Secondly, dues to the mobility limitations of some participants–such as inability to lie down and rotate from supine to prone position, we were unable to collect TMG data from them. We could speculate that these participants were indeed sarcopenic and therefore the results presented in this study could underestimate sarcopenia-related changes in contractile parameters. Additionally, our MANCOVA analysis yielded small effect sizes, indicating that only a limited proportion of the variance in the TMG parameters is explained. This suggests that, while in between-group differences are significant, their practical and clinical impact should be further validated in future studies.

## Conclusion

In conclusion, TMG-derived muscle contractile parameters showed differences between sarcopenic and non-sarcopenic individuals, although these differences were not uniform across all muscles. Overall, weak to moderate but consistent associations were obsereved between TMG-derived paramters and sarcopenia classification tests, with muscle- and sex-specific patterns. All four TMG parameters were interchangeably correlated with sarcopenia classification tests. Importantly, the direction of these associations was uniform, indicating a more favorable contractile characteristics were consistently related to better sarcopenia-related performance outcomes. While differences between sarcopenia classifications were obsereved, the diagnostic relevance of these findings remains to be established. Given the small effect sizes, the present results should be interpreted as exploratory. Future studies are needed to determine the clinical utility of TMG-derived parameters and to evaluate their role in sarcopenia assessment across different classifications.

## Data Availability

The original contributions presented in the study are included in the article/supplementary material, further inquiries can be directed to the corresponding author.
