# Supplementary material for: Tensiomyography-derived contractile parameters in sarcopenic and non-sarcopenic older adults
Source: Front Aging. 2026 Feb 4;7:1719152. doi: 10.3389/fragi.2026.1719152 (PMC12913555; doi:10.3389/fragi.2026.1719152)

Figure 1: Distribution of physical performance tests split by sarcopenia severity classified by EWGSOP2


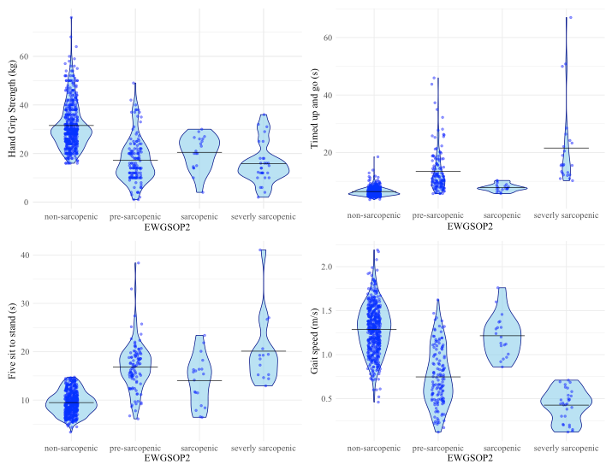


Figure 2: Distribution of physical performance tests split by sarcopenia severity classified by SDOC


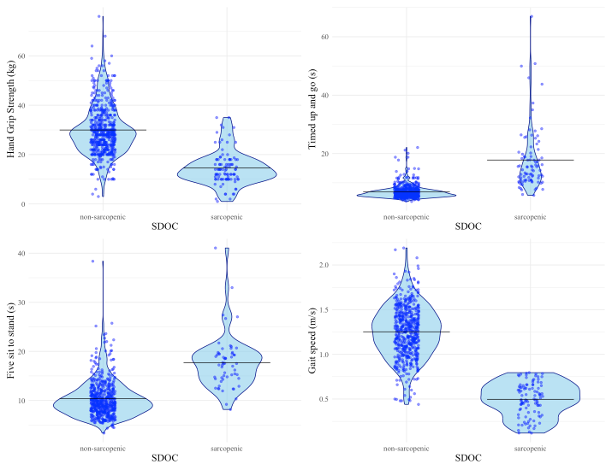


Figure 3: TMG-derived parameters distribution split by sarcopenia classifications (SDOC and EWGSOP2) in vastus lateralis


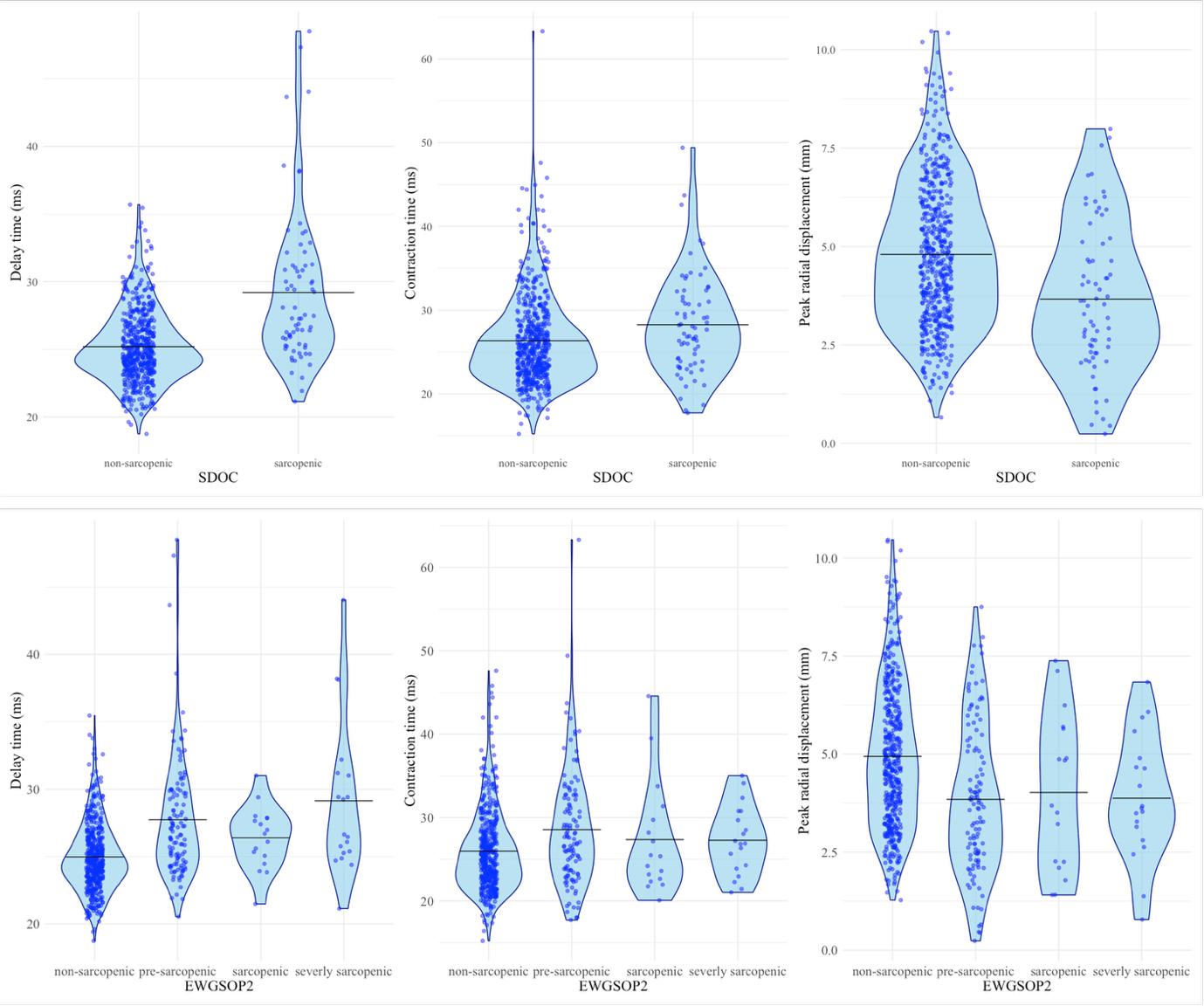


Figure 4: Figure 3: TMG-derived parameters distribution split by sarcopenia classifications (SDOC and EWGSOP2) in biceps femoris


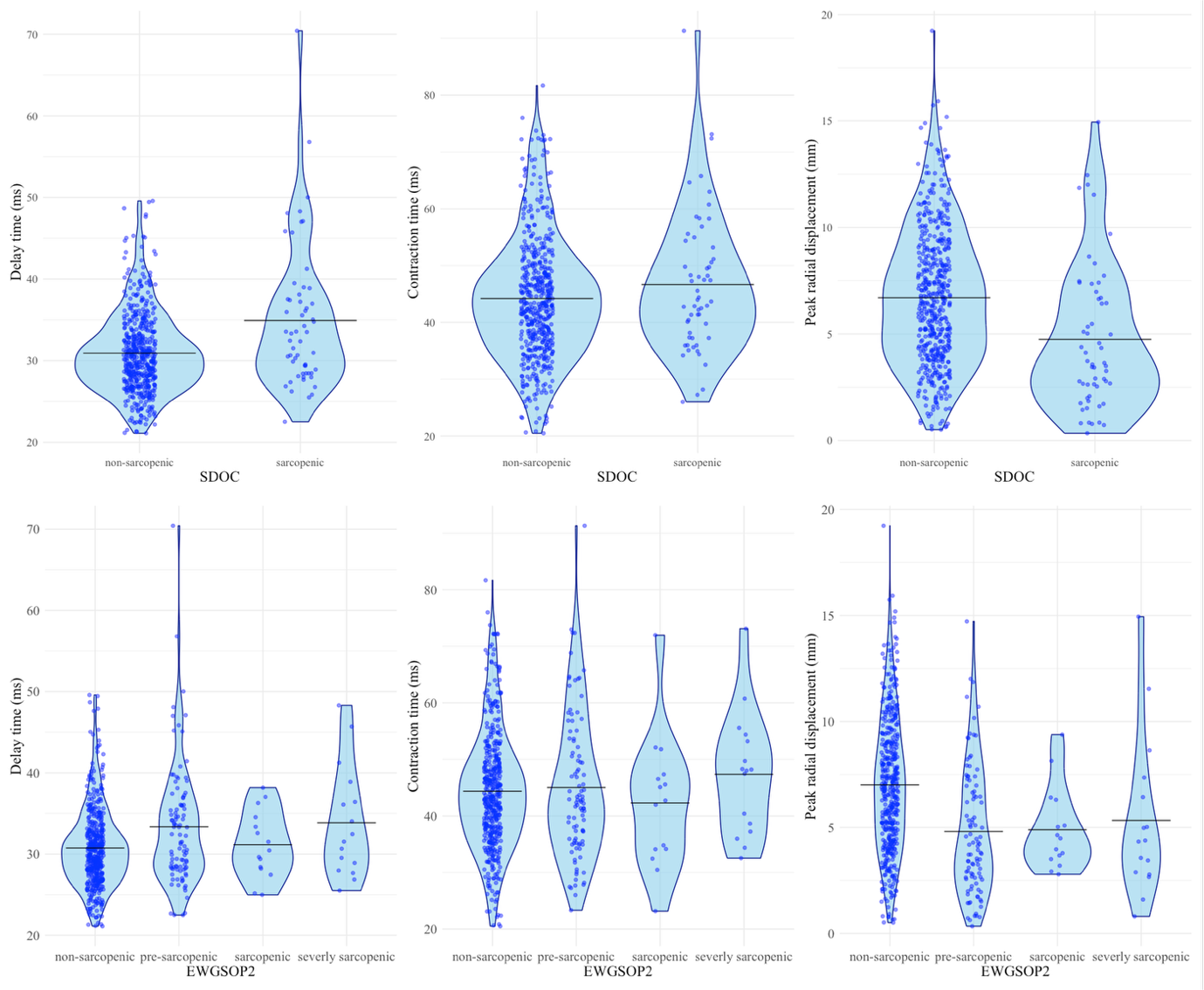


Figure 5: Figure 3: TMG-derived parameters distribution split by sarcopenia classifications (SDOC and EWGSOP2) in gastrocnemius medialis


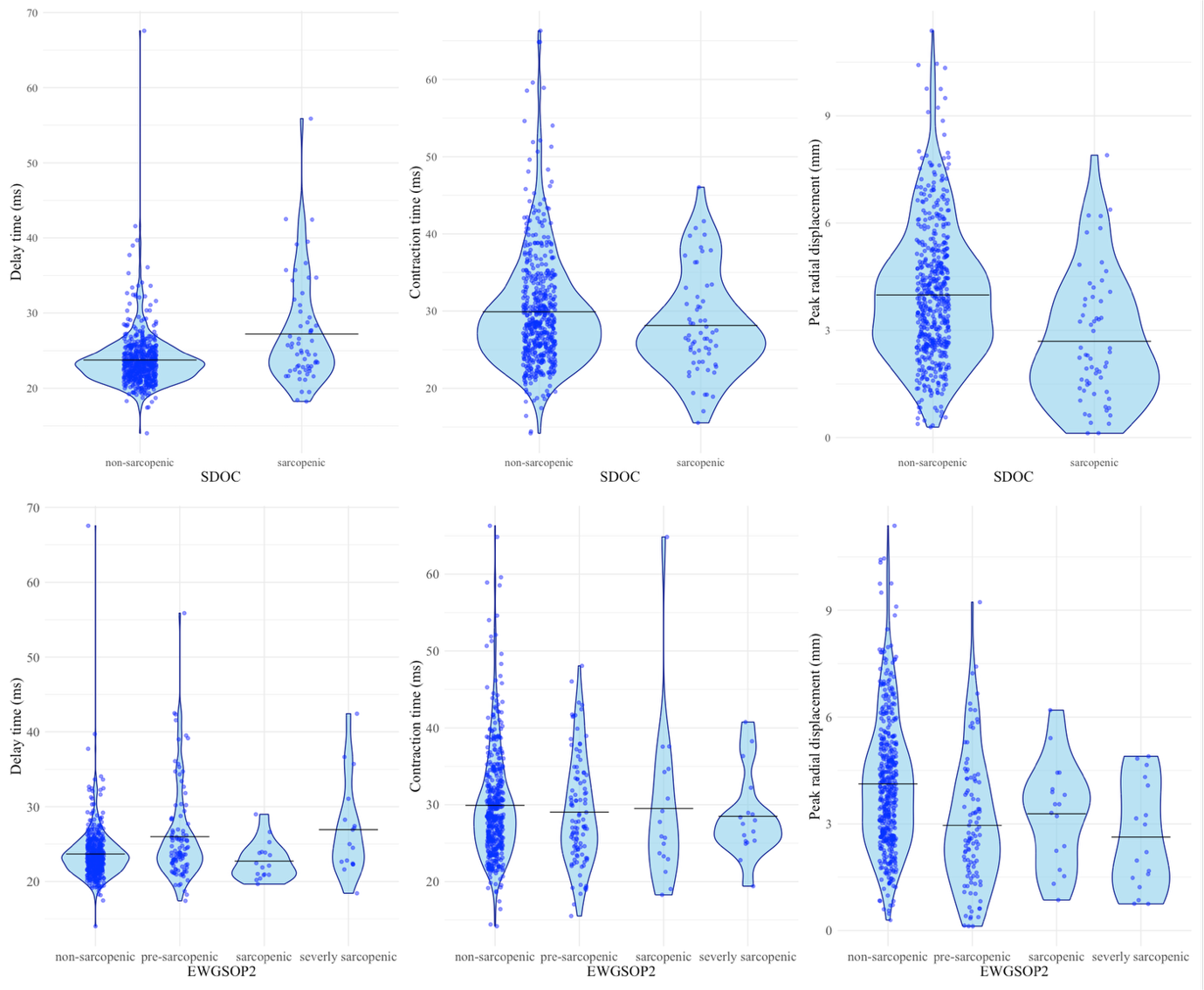

Supplement: Supplementary file 1 [file Supplementaryfile1.docx]
